# Correction: Inactive matrix gla protein plasma levels are associated with peripheral neuropathy in Type 2 diabetes

**DOI:** 10.1371/journal.pone.0232996

**Published:** 2020-05-05

**Authors:** Anne-Caroline Jeannin, Joe-Elie Salem, Ziad Massy, Carole Elodie Aubert, Cees Vemeer, Chloé Amouyal, Franck Phan, Marine Halbron, Christian Funck-Brentano, Agnès Hartemann, Olivier Bourron

The fifth author's name is spelled incorrectly. The correct name is: Cees Vermeer. The correct citation is: Jeannin A-C, Salem J-E, Massy Z, Aubert CE, Vermeer C, Amouyal C, et al. (2020) Inactive matrix gla protein plasma levels are associated with peripheral neuropathy in Type 2 diabetes. PLoS ONE 15(2): e0229145. https://doi.org/10.1371/journal.pone.0229145
