# Correction: Correction: Inactive matrix gla protein plasma levels are associated with peripheral neuropathy in Type 2 diabetes

**DOI:** 10.1371/journal.pone.0248441

**Published:** 2021-03-04

**Authors:** The PLOS ONE Staff

The fifth author’s name is spelled incorrectly in the byline of the correction published on May 5, 2020. The publisher apologizes for the error. The correct name is: Cees Vermeer. The correct citation is: Jeannin A-C, Salem J-E, Massy Z, Aubert CE, Vermeer C, Amouyal C, et al. (2020) Correction: Inactive matrix gla protein plasma levels are associated with peripheral neuropathy in Type 2 diabetes. PLoS ONE 15(5): e0232996. https://doi.org/10.1371/journal.pone.0232996.
