# Supplementary material for: Inactive matrix gla protein plasma levels are associated with peripheral neuropathy in Type 2 diabetes
Source: PLoS One. 2020 Feb 24;15(2):e0229145. doi: 10.1371/journal.pone.0229145 (PMC7039520; doi:10.1371/journal.pone.0229145)
Supplement: S1 Table — (DOCX) [file pone.0229145.s001.docx]

**S1 Table: Correlations between dp-ucMGP and coronary arterial disease and other micro-vascular complications of diabetes.**

|  | r | p-value |
| --- | --- | --- |
| coronary arterial disease | 0.051 | 0.48 |
| Lasered retinopathy | -0.134 | 0.06 |
| eGFR (MDRD) | **-0.377** | **< 0.0001** |
| Albuminuria* | **-0.268** | **0.0001** |

*r is calculated by Pearson correlation test (dp-ucMGP being normally distributed). Correlations are significant if p<0.05. Significant results are presented in bold. * defined by urinary albumin/creatinine ratio >3 mg/mmol.*
