# Supplementary material for: Inactive matrix gla protein plasma levels are associated with peripheral neuropathy in Type 2 diabetes
Source: PLoS One. 2020 Feb 24;15(2):e0229145. doi: 10.1371/journal.pone.0229145 (PMC7039520; doi:10.1371/journal.pone.0229145)
Supplement: Attachment — Submitted filename: renamed_5a3c2.docx [file pone.0229145.s002.docx]

Response to Reviewers.

We thank the reviewers for carefully reading our article. As requested we have highlighted changes made from the original version in a separate file labeled 'Revised Manuscript with Track Changes'. An unmarked version of our revised paper without tracked changes was also made. This file was uploaded as separate file and labeled 'Manuscript'.

1. The title of the manuscript “Inactive Matrix Gla Protein serum levels….”, while in the methodology section the authors state that plasma dp-ucMGP levels were measured. Which is it?

*Thanks to the reviewer for pointing out this error. We changed the title as follow: « Inactive Matrix Gla Protein ~~serum~~ plasma levels are associated with peripheral neuropathy in Type 2 Diabetes »*

2. Since dpucMGP has been repeatedly associated with vascular calcification and cardiovascular disease and diabetes complications such as diabetic nephropathy, it would be interesting to show any associations between circulating dpucMGP and coronary arterial disease, retinopathy , eGFR and albuminuria.

*In our cohort, we confirmed that dp-ucMGP was significantly associated with eGFR .*

|  | r | p-value |
| --- | --- | --- |
| coronary arterial disease | 0.051 | 0.48 |
| Lasered retinopathy | -0.134 | 0.06 |
| eGFR (MDRD) | **-0.377** | **< 0.0001** |
| Albuminuria* | **-0.268** | **0.0001** |

***S1 Table.*** *correlations between dp-ucMGP and coronary arterial disease and other micro-vascular complications of diabetes. r is calculated by Pearson correlation test (dp-ucMGP being normally distributed). Correlations are significant if p<0.05. Significant results are presented in bold. * defined by urinary albumin/creatinine ratio >3 mg/mmol.*

*We have included, line 273 page 11, in the discussion, a paragraph about these data:*

There is some data showing that dp-ucMGP is associated with several micro and macro-vascular complications of diabetes, including diabetic nephropathy, retinopathy, vascular stiffness and vascular calcification(39-41). Although dp-ucMGP has been repeatedly associated with vascular calcification and cardiovascular disease, dp-ucMGP is not associated in our study with coronary arterial disease (S1 table)(39, 41). As observed by others, dp-ucMGP is associated negatively with eGFR estimated by MDRD and with albuminuria (S1 table) (39, 40). However we don’t find any association between dp-ucMGP and retinopathy treated by laser (S1 table) despite some data suggesting that dp-ucMGP could be a marker of retinal health(42). Additional studies are needed to explore specifically these associations in patients with diabetes.

*We have added the S1table in supplementary data.*

3. In the discussion part, the authors state that “Insulin use in Type 2 Diabetes reflects indirectly diabetes duration and poor glycemic control”. Given the fact that BMI includes height and insulin use reflects diabetes duration and HBA1c, how can the authors eliminate the possibility of statistical overlapping in multivariate analysis, where all these variables were included?

*We agree with the reviewer that this statement in the discussion is misleading and we dropped it.*

*None of these parameters (BMI, diabetes duration and HbA1C level) were associated with presence of neuropathy in univariate analysis, as compared to insulin use and height which were (see table below).*

| Characteristics | Total cohort | Neuropathy (NDS≥6) | Without neuropathy (NDS<6) | p-value |
| --- | --- | --- | --- | --- |
| BMI (Kg/m2) | 29,16±5.3 | 30,23±5.5 | 28,97±5.2 | ns |
| Diabetes duration, years | 14.6±9.3 | 14.6±10.2 | 14.6±9.2 | ns |
| HbA1c, mmol/mol | 61.8±16.2 | 66.6±20.5 | 60.9±15.2 | ns |
| HbA1c, % | 7.8±1.5 | 8,2±1.9 | 7.7±1.4 | ns |
| **Height (cm)** | 170±8 | 173±7 | 169±8 | **0.009** |
| **Insulin treatment, n(%)** | 94 (47.5) | 24(77.4) | 70(41.9) | **0.0003** |

*We have modified the paragraph corresponding (line 216 p9) as follow:*

We don’t find any significant association between BMI and neuropathy in our study, probably because BMI formula includes height, which is maybe a more important marker of diabetic peripheral neuropathy due to the length-dependent presentation of this neuropathy(20). Furthermore, BMI is not associated with neuropathy in the DIACART study also probably because majority of the patients included were overweight or obese (mean BMI 29.16+/-5.3 Kg/m^2^). ~~Insulin use in Type 2 Diabetes reflects indirectly diabetes duration and poor glycemic control. So that is probably why it is associated with diabetic neuropathy in the DIACART study.~~ Although insulin is considered as a neurotrophic factor and although low-dose insulin can have beneficial effects on diabetic neuropathy, insulin use is associated with diabetic neuropathy in the DIACART study(21). Retinopathy and nephropathy are usual comorbidities of diabetic neuropathy, explaining their association in this study(4). ~~BMI was not associated with neuropathy in the DIACART study probably because majority of the patients were overweight or obese (mean BMI 29.16+/-5.3 Kg/m~~^~~2~~^~~).~~

4. The discussion part should be enriched with data showing that dpucMGP has been associated with several micro and macro vascular complications of diabetes, including diabetic nephropathy, retinopathy, vascular stiffness and calcification (suggested references to be added: doi:10.3390/ijms20030628, doi: 10.1038/s41598-018-33257-6, doi: 10.1093/ajh/hpy079, doi: 10.1159/000443426 and doi: 10.1016/j.jdiacomp.2017.06.012)

*We have added these references in the discussion.*
